# Efficacy of a culturally tailored cognitive-behavioural intervention for Ethiopian children with haematological malignancies: study protocol for randomised controlled trial

**DOI:** 10.1186/s13063-022-06768-x

**Published:** 2022-09-27

**Authors:** Tenaw Gualu Melesse, Janita Pak Chun Chau, William Ho Cheung Li

**Affiliations:** 1 Nethersole School of Nursing, Faculty of Medicine, The Chinese University of Hong Kong, Hong Kong, Hong Kong; 2 Department of Paediatrics and Child Health Nursing, College of Health Sciences, Debre Markos University, Debre Markos, Ethiopia

**Keywords:** Cognitive-behavioural intervention, Children, Haematological malignancy

## Abstract

**Background:**

Paediatric cancer patients often experience anxiety and depression. Evidence suggests that cognitive-behavioural interventions may help reduce anxiety and depression in children undergoing cancer treatment. However, only a few studies evaluated its impact on the psychological well-being and quality of life of paediatric cancer patients globally. In Ethiopia, there has been no published study to date. Thus, this trial aims to evaluate the efficacy of a culturally tailored cognitive-behavioural intervention for Ethiopian children with haematological malignancies receiving chemotherapy.

**Methods:**

A single-blinded, parallel-group, two-arm, repeated measure randomised controlled trial will be conducted. Eighty children aged 8 − 18 years with haematological malignancy receiving chemotherapy will be recruited and randomly assigned to experimental or control groups. The experimental group will receive five sessions of introducing cognitive-behavioural intervention, identifying and modifying maladaptive thoughts and behaviour, behavioural activation, practising deep breathing exercises, reassessing goals or treatment plans, and encouraging participants to maintain changes. Each session will be conducted face-to-face for 30–35 min a week. The control group will receive usual care. The outcomes will be measured at baseline, post-intervention, and one month after the intervention using the Revised Child Anxiety and Depression Scale and Paediatric Quality of Life Inventory Generic Core Score 4.0.

**Discussion:**

The findings of this study will provide evidence to support the integration of culturally effective cognitive-behavioural intervention strategies into paediatric oncology practice and thus, add new knowledge to the literature and help improve the care of children with haematological malignancies receiving chemotherapy. If the cognitive-behavioural intervention is shown to be effective and culturally acceptable, it will provide evidence to include the intervention as a standard of care in paediatric haematology/oncology.

**Trial registration:**

ClinicalTrials.gov NCT05270655. Registered on March 8, 2022.

**Supplementary Information:**

The online version contains supplementary material available at 10.1186/s13063-022-06768-x.

## Background

Haematological malignancies include malignancies of the blood, bone marrow, and lymph nodes. Leukaemia, lymphoma, and myeloma are the most common types of paediatric cancer all over the world [1–3]. In Ethiopia, evidence shows that more than 50% of childhood cancers are haematological malignancies [4, 5].

The course of childhood cancer causes various stressful symptoms among children [6]. The diagnosis, course of treatment such as painful medical procedures and chemotherapy side effects, and challenges in social life such as missing the opportunity to play with friends, separation from family members, and school absence are major stressors among children [7, 8]. Although most paediatric cancer patients experience various psychological problems, anxiety and depression are among the most frequently reported problems [9, 10]. Childhood cancer also significantly impedes their quality of life (QOL) [11–16].

Poorly managed anxiety and depression significantly impact QOL and survival status [17, 18]. Unmanaged anxiety predicts fatigue [7], anticipatory pain, nausea and vomiting during the subsequent medical procedures [19] and increases off-therapy anxiety [20]. Poorly treated depression impairs relationships and psychological functioning [21], increases the burden of symptoms and mortality [9], and affects motivation to change behaviour and evidence suggests these patients are less likely to adhere to treatment [22]. Non-adherence to treatment including chemotherapy decreases prognosis increases relapse [23, 24], morbidity and hospital readmissions [17] and treatment costs, and has a negative impact on QOL [25]. Likewise, poor QOL is associated with emotional, behavioural, and social problems such as difficulty establishing or maintaining friendships and poor school performance [26].

Psychosocial problems, such as anxiety and depression in children with cancer are given little attention and are poorly managed [27] as more emphasis is given to the physical aspect of the illness. Thus, a tailored psychosocial intervention that has been found to be effective should be incorporated into the current medical treatment [9]. Psychosocial interventions including music-based interventions [28–30], art therapies [31–34], play-based interventions [35–37] and cognitive-behavioural interventions (CBI) [38–40] show promising effects in improving the health outcomes of children with cancer. Among psychosocial interventions, CBI is most frequently recommended to manage anxiety and depression in paediatric cancer patients due to its effectiveness and feasibility [38–41].

Evidence suggests that CBI helps improve psychosocial and health-related outcomes in children with cancer [42–46]. However, as most previous studies were conducted in developed countries, adopting and applying CBI approaches and strategies in other countries might be ineffective. In addition, in most studies, the theory guiding CBI development was not identified. Thus, developing and evaluating a theory-guided CBI in a different country such as Ethiopia is essential.

CBI is a structured and tailored psychotherapeutic intervention based on cognitive and behavioural approaches [47, 48], and helps to modify distorted thinking and beliefs, reduce maladaptive behaviour, and increase coping skills [49]. CBI enables patients to manage their problems independently and to maintain behavioural changes [47, 50].

In this study, we developed a CBI for children with haematological malignancies guided by Beck’s cognitive model [50]. The model describes the associations among cognition, emotion, and behaviour [47]. Distorted thoughts and unhelpful coping strategies often cause emotional problems such as anxiety and depression [51–53]. For instance, in Ethiopia, most patients experience extreme stress as they believe that there is no hope of childhood cancer being cured with modern medicine. They abandon treatment because they believe religious and spiritual therapies, or alternative forms of treatment such as visiting traditional healers and using holy water, are more effective [54, 55]. Thus, CBI strategies help identify and modify distorted thoughts and unhelpful behaviour [48] and promote the development of positive coping strategies, and cognitive and behavioural adaptation [47, 56]. Positive changes in the cognitive process and development of positive coping behaviour help reduce stressful feelings such as anxiety and depression and, in turn, could improve psychosocial functioning and QOL [57].

## Methods

### Objectives

The objectives of this study are to evaluate whether a theory-based CBI achieves (1) reduced anxiety and depression and (2) improved quality of life post-intervention in the experimental group compared to the control group receiving usual care.

### Study design

This trial is designed as a multi-centre, assessor-blinded, two-arm parallel-group, repeated measure randomised controlled trial (RCT). The protocol for this trial was registered at ClinicalTrials.gov (NCT05270655). A multi-centre trial helps recruit adequate and representative participants and thus, improves the generalisability of the study findings [58].

**Fig. 1:**
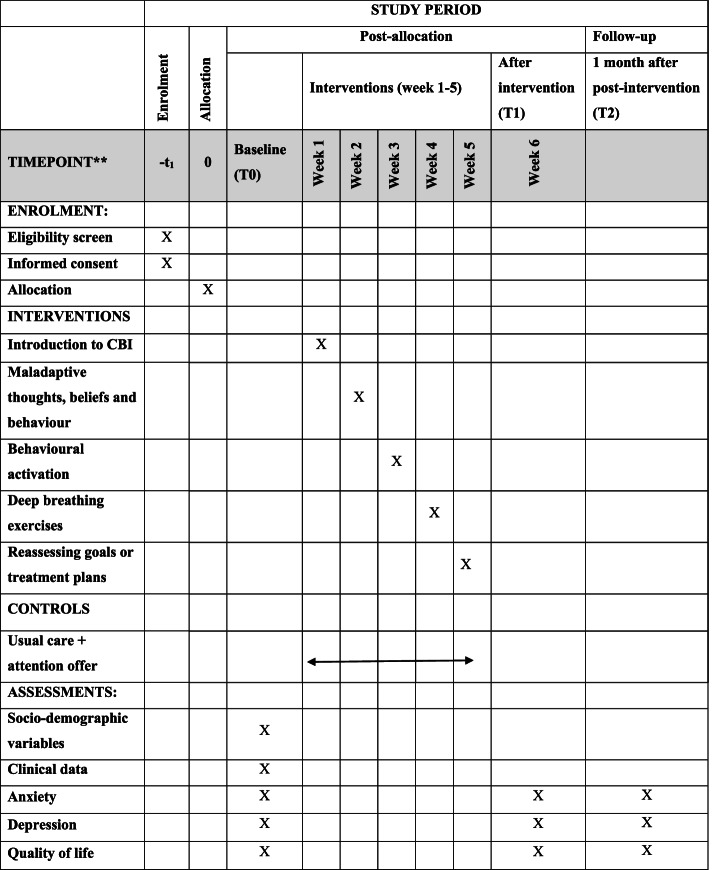
SPIRIT figure

The schedule of enrolment, interventions and assessments of the study protocol follows the Standard Protocol Items: Recommendations for Interventional Trials (SPIRIT) Figure (Fig. 1) and SPIRIT Checklist (Additional File 1) [59].

### Study setting

Participants will be recruited from the paediatric haematology-oncology wards in two university-affiliated specialised hospitals. These hospitals are among the largest specialised hospitals providing medical services to many children with cancer in Ethiopia.

### Participants

Participants (1) aged 8 to 18 years old, (2) diagnosed with haematological malignancy, (3) undergoing chemotherapy, (4) able to communicate in Amharic, and (5) with written parental consent and oral child assent will be recruited. Participants (1) with evidence of developmental, psychological and/or psychiatric illness or problems**,** (2) unable to collaborate in the study due to acute illness, and/or (3) having hearing or speech problems that could affect the study process will be excluded.

### Sample size

The sample size was estimated using the G*Power (version 3.1.9.7) sample size calculator based on the effect size of CBI on anxiety, the primary outcome of this study. Evidence shows that the effect size of CBI ranges from 0.7 to 3.9 [43, 45, 46, 60]. Using the smallest effect size of 0.7, 80% power, 5% significance level and a potential 15% attrition rate, 80 participants (40 per group) will be recruited.

### Participant recruitment

A research assistant (RA) in each hospital with the help of nurses in the paediatric oncology ward will approach children and their parents to identify their eligibility, explain the study procedures, and provide a detailed information sheet. For participants who agree to join the study, the RA will obtain written parent consent and oral child assent, collect baseline clinical and demographic data, and provide the participants with an appointment card.

### Randomisation

Participants will be stratified based on their age groups, i.e. ≤ 12 years and ≥ 13 years in each hospital [61]. Evidence shows that age influences the level of anxiety, depression, and QOL [11, 62, 63]. Stratified randomisation balances comparability between the groups and increases the power of the study [64]. The principal investigator will generate a computerised random sequence with a 1:1 allocation using a block size of 4 and 6 for each stratum of each hospital.

### Allocation concealment

Participants, research assistants (RAS) and intervention providers would not know to which group the participants will be assigned. The allocation sequence will be concealed in a sequentially numbered, sealed, and opaque envelope until treatment assignment.

### Blinding

The outcome evaluators are paediatric nurses who are not working in the study hospitals and will be blinded to the study groups. To ensure blinding, participants will be advised not to disclose their group assignment to outcome evaluators. However, due to the nature of the intervention, it is not possible to blind participants and intervention providers. This is an open-label trial with only patient-outcome assessors being blinded so emergency unblinding will not occur.

### Intervention procedures

Guided by Beck’s cognitive model [50], the content and dose of CBI are determined by reviewing international guidelines on CBI [48, 50, 65], and the findings of the systematic review and meta-analysis [66]. Beck’s model describes how thought, feeling, and behaviour interact. Hence, CBI helps identify and modify maladaptive thoughts and behaviour and develops coping skills. Intervention content and strategies for the experimental group will be tailored to the child’s age and health conditions and include psychoeducation, guided discovery or Socratic questioning, discussion, drawing/painting or writing, and play supplemented with home-based practices.

### Experimental group

The experimental group will receive five weekly face-to-face sessions (30–35 min each). The intervention will be delivered by two psychologists with master’s degree who have clinical and research experience in CBI. The contents include (1) an introduction to CBI, (2) identifying, evaluating, and challenging maladaptive thoughts, beliefs and behaviour, (3) behavioural activation, (4) practising deep breathing exercises, and (5) reassessing goals or treatment plans, and encouraging participants to maintain changes.

The intervention will be delivered in the hospital before the children undergo chemotherapy to avoid excessive fatigue and increase their engagement with the interventions. Additionally, to establish a strong family support system and enhance intervention delivery, parents or primary caregivers of the child will be invited to attend the introduction of the first session, the summary of each session, and whenever else necessary. However, the role of the parents/caregivers is only to facilitate the intervention delivery, such as providing information when needed.

To enhance the retention rate, interventions will be delivered during participants’ medical appointments at the hospitals. In addition, each participant will be provided with an appointment card containing the date and time of the next CBI appointment, and the RA will remind them with a phone call 2 days before the next appointment. The participants will continue receiving the usual psychosocial care provided by the nurses in the paediatric oncology ward.

### Control group

The control group will receive the usual psychosocial care such as information and health education about the course of the illness and treatments provided by the nurses in the paediatric oncology ward. Additionally, the RA will offer them attention every week for 30 min about treatment adherence and other concerns. Parents will be invited to attend if they wish to do so.

### Outcomes and measurements

#### Primary outcome

Anxiety will be assessed using the anxiety subscale of the self-report 25-items Revised Child Anxiety and Depression Scale (RCADS) (α = 0.91 for anxiety scale) in the original study [67], before the intervention (T0, baseline), immediately after the intervention (T1, at 6 weeks) and one month after completion of the intervention (T2, at 10 weeks). In this study, the Amharic version of the RCADS will be used. The Amharic version of the scale exhibited satisfactory validity and reliability in our other study (*α* = 0.95 and *α* = 0.96 for the anxiety sub-scale and total scale respectively) [unpublished data]. Each item will be rated on a four-point Likert scale (0–3), and the total raw anxiety score ranges from 0 to 45 [68]. To calculate the total anxiety score, the raw scores will be converted into *T*-scores, with *T*-scores of ≥ 65 to 70 and ≥ 70 being borderline and above the clinical threshold, respectively [69]. A higher anxiety score corresponds to higher levels of anxiety.

#### Secondary outcomes

For the secondary outcomes, depression and QOL will be assessed at T0, T1, and T2. The level of depression will be assessed using the depression subscale of the RCADS (*α* = 0.80 for the depression scale in the original study) [67], and *α* = 0.94 for the Amharic version in our study [unpublished data]). A higher score reflects higher depression.

QOL will be measured using a child-report PedsQL™ 4.0 GCS for ages (8 − 12 and 13 − 18 years) [70]. The Amharic version of the scale demonstrated good reliability and validity in our other study (α = 0.96) [unpublished data]. To calculate the total score, each item will be scored on a five-point Likert scale (0–4), and the 0 − 4 scale items will be reversely scored and linearly transformed to 0–100 [71]. A higher score indicates high QOL.

#### Other measures

**Fig. 2:**
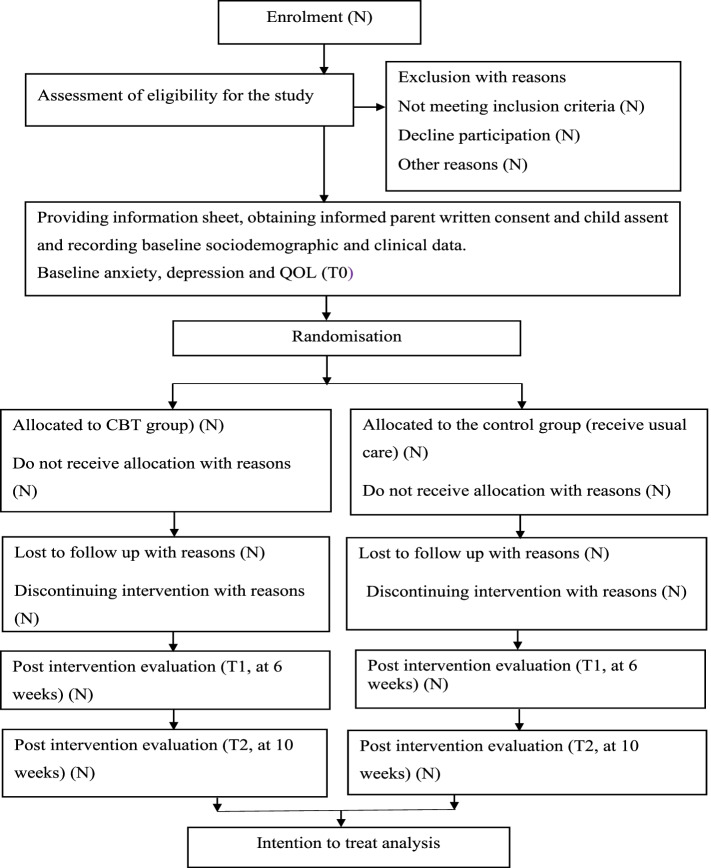
Study procedures adapted from consolidated standards of reporting trials (CONSORT) flow diagram [72]

Participants’ demographic and clinical characteristics will be recorded at baseline using a demographic and clinical datasheet. The overall study process is summarised in Fig. 2.

### Data analysis

The analysis will be conducted using Statistical Package for the Social Sciences (IBM SPSS statistics 26). To check discrepancies in data entry, the principal investigator and a RA will independently enter the coded data twice into SPSS. While categorical data will be summarised using frequencies and percentages, continuous data will be summarised using means, standard deviations, median and interquartile ranges depending on normality distribution. Depending on the types of data and normality distribution, the characteristics of the groups will be compared using independent t-tests, Mann–Whitney U tests, Chi-square tests, and Fisher’s exact tests. Outcome analysis will be conducted as an intention-to-treat analysis. Missing value analyses will be conducted to determine the need for multiple imputations. A generalised estimation equation (GEE) model will be applied to determine the intervention effect between the groups in terms of a group, time, and group*time interaction. Variability in effect will be assessed by performing a sub-group analysis. Given that this trial is studying a low-risk or no-risk intervention, there are no stopping rules or plans for interim analyses. The significance level will be determined at 5% (two-sided) for all statistical tests.

### Oversight and monitoring

The coordinating centres are composed of the principal investigator and the research team (clinical psychologists and research assistants) and are responsible for the day-to-day organisational support for the trial. The PI will meet them every 2 weeks to discuss the trial progress and any issues that arise during the trial. The steering committee includes the PI, site supervisors (paediatric haematologists-oncologists), and a psychiatrist who will meet every 2 months. However, as the intervention of this trial is a low or no-risk intervention, there is no data monitoring committee. To review the trial conduct, the study team will meet every 2 weeks led by the PI throughout the study. The steering committee will also meet every 2 months. Finally, a progress report will be submitted to the Ethics committees at the end of the trial.

## Discussion

Most previous trials that evaluated CBI in paediatric oncology focused on parent or caregiver outcomes. Other studies that evaluated child outcomes were conducted in developed countries. However, CBI strategies in these countries might not be acceptable for patients with diverse backgrounds and the effects of the interventions may differ when adopted and applied in different cultural contexts such as Ethiopia. In addition, most of these trials had methodological limitations and the authors recommended conducting further rigorous trials [66].

This RCT can be considered the first to evaluate CBI for children with cancer in Ethiopia. It aims to evaluate the effects of a theory-based and age-appropriate cognitive behavioural intervention to improve anxiety, depressive symptoms, and QOL of children with haematological malignancies. Thus, if the intervention is found to be effective and acceptable, it will pave the way for the integration and implementation of CBI into paediatric haematology-oncology to improve the psychological symptoms and QOL of paediatric cancer patients.

### Trial status

Protocol version 2, September 2022.

Participant recruitment commenced in May 2022 and the study will be completed in November 2022.

## Supplementary Information

**Additional file 1. **

## Data Availability

The final data set for this trial can be obtained from the corresponding author upon reasonable request.

## Abbreviations

CBI: Cognitive-behavioural intervention
CUHK-NTEC CREC: Joint Chinese University of Hong Kong-New Territories East Cluster Clinical Research Ethics Committee
QOL: Quality of life
RCADS: Revised Child Anxiety and Depression Scale
SPIRIT: Standard Protocol Items: Recommendations for Interventional Trials
SPSS: Statistical Package for the Social Sciences
